# Family-to-Work Interface and Workplace Injuries: The Mediating Roles of Burnout, Work Engagement, and Safety Violations

**DOI:** 10.3390/ijerph182211760

**Published:** 2021-11-09

**Authors:** Oi Ling Siu, Ting Kin Ng

**Affiliations:** Department of Applied Psychology, Lingnan University, 8 Castle Peak Road, Tuen Mun, New Territories 999077, Hong Kong; ngtingkin@gmail.com

**Keywords:** family-to-work interface, workplace injuries, burnout, work engagement, safety violations

## Abstract

Past research has primarily investigated the role of the negative side (family-to-work conflict; FWC) of the family-to-work interface in workplace safety outcomes and neglected the positive side (family-to-work enrichment; FWE). Moreover, the mechanism underlying the relation between the family-to-work interface and workplace safety has not been well studied. From the perspectives of the job demands-resources model as well as conservation of resources theory, this study endeavors to extend the current literature on workplace safety by evaluating the mediating roles of burnout, work engagement, and safety violations in the associations of FWC and FWE with workplace injuries. Two-wave longitudinal survey data were obtained from 233 Chinese employees in two high-risk industries (nursing and railways). The hypothesized longitudinal mediation model was analyzed with the structural equation modeling technique. It was revealed that the association of FWE with workplace injuries was mediated by work engagement and then safety violations. Burnout was found to mediate the association of FWC with workplace injuries. Safety violations were also found to mediate the association of FWC with workplace injuries. The present findings offer insights into the underlying mechanisms by which the family-to-work interface influences workplace injuries.

## 1. Introduction

Occupational safety has been a central concern for organizations, especially those in high-risk industries. About 250 million work-related accidents and over 300,000 work-related fatal injuries occur worldwide annually, resulting in a large economic loss of 1.8–6% GDP [1]. It has been demonstrated that more than half of occupational accidents can be attributed to human error [2]. However, according to risk-taking research, there are various factors (e.g., self-efficacy, meeting organizational purpose) that make an employee willing to take risks and engage in unsafe tasks [3].

**Figure 1:**
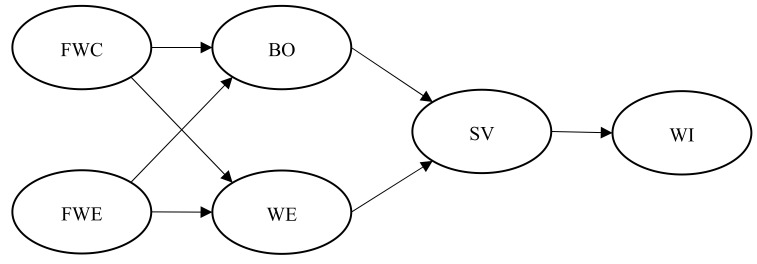
The theoretical model. FWC = family‐to‐work conflict; FWE = family‐to‐work enrichment; BO = burnout; WE = work engagement; SV = safety violations; WI = workplace injuries.

Scholars have investigated the psychosocial determinants of workplace safety, including safety climate, personality traits, and job attitudes [4,5,6,7,8,9]. Despite substantial progress made in this line of research, scholars have neglected the roles of the work–family interface in workplace safety [7,8,10,11]. In Nahrgang et al.’s [8] meta-analysis, the work–family interface (connections between work and home/non-working life) was not incorporated in the theoretical model (see Figure 1, p. 72). Family and work are two key components of employees’ lives, and the interaction between them does influence employees’ work behavior [12,13,14].

Conservation of resources (COR) theory holds that the interference between the familial and work roles may engender a loss of psychological resources [15,16], leading to detrimental consequences on employees’ positive work behaviors [17,18]. By contrast, if work and family life are mutually beneficial, psychological resources (e.g., positive emotions) are gained and subsequently promote employees’ positive work behaviors [19]. The work–family interface may thus be an important antecedent of employees’ workplace safety outcomes, yet, in particular, studies on the family-to-work direction have been relatively rare [11,20]. This study focused on how the family-to-work interface relates to workplace injuries, which were operationalized as the self-reported rate of injuries at the workplace [21,22,23] in this study.

The theoretical contributions of the current study are threefold. First, we take into account employees’ experiences of the family life domain to better understand how they influence workplace safety. Although recent studies have recognized the role of the work–family interface in employees’ safety attitudes [10,24], how it might affect workplace safety outcomes (e.g., workplace injuries) is not well understood.

Second, we investigate the influences of both the negative side (family-to-work conflict; FWC) as well as the positive side (family-to-work enrichment; FWE) of the family-to-work interface on workplace safety. Studies have primarily investigated the role of FWC in workplace safety [10,11] and neglected FWE. However, some studies have indicated that FWE benefits job performance in general [25,26,27]. Moreover, recent research has pointed out that FWE was related to better work-related attitudes and behaviors [25,28,29]. The current study examined the influences of both FWC and FWE on workplace injuries.

Third, the mechanism underlying the relation between the family-to-work interface and workplace safety has not been well understood. This study seeks to offer insights into why the family-to-work interface can have negative and positive impacts on workplace injuries. Specifically, we proposed a strain process underlying the unfavorable influence of FWC and a motivational process underlying the favorable influence of FWE on workplace injuries. From the perspectives of the job demands-resources (JD-R) model [30,31] and COR theory [15,16] and echoing the health impairment versus motivational processes reviewed by Nahrgang et al. [8], we investigated the mediating roles of burnout, work engagement, and safety violations in the relations of FWC and FWE with workplace injuries among nurses and railway workers. Nurses and railway workers are shift workers who are likely to be affected by work–family interference.

### 1.1. Family-To-Work Conflict

FWC is experienced when an employee’s family responsibilities impede work-related performance [17]. FWC is especially prevalent among shift workers (e.g., nurses, railway workers), whose irregular work schedule may pose difficulties for employees to deal with both family and work demands.

The JD-R model and COR theory may explain how FWC influences safety outcomes. The JD-R model is a theoretical framework that explains how job characteristics (job demands and job resources) influence work outcomes through two distinct processes (health impairment and motivational processes) [30,31]. This model identifies two distinct classes of work-related factors. First, job demands refer to the facets of work that need effort and have mental and physical costs. Second, job resources refer to the facets of work that facilitate goal attainment, mitigate job demands and related costs, and provide opportunities for personal development [30,31].

Job demands tend to elicit a health impairment process exhausting individuals’ physical and psychological resources and hence result in burnout [30,31], which refers to a condition of physiological and psychological exhaustion resulting from emotionally demanding circumstances [32]. Burnout encompasses three components, including emotional exhaustion (a state of depletion of mental energy), cynicism or depersonalization (a negative attitude toward work), and low professional efficacy (low sense of personal accomplishment at work) [32]. Moreover, hindrance job demands (e.g., role conflict) are barriers that hinder effective performance and therefore lead to a decrease in work engagement [30,31], which refers to a satisfying condition with high levels of physical and mental energy (vigor), involvement in work (dedication), and concentration on work (absorption) [33]. FWC can be considered a kind of hindrance job demand. In this light, it is plausible to hypothesize that FWC will lead to elevated burnout and decreased work engagement.

Moreover, COR theory argues that preserving resources is a fundamental human need [15,16]. Individuals experience strain when they encounter the threat of losing resources [15]. FWC, as a hindrance stressor, consumes individuals’ valuable resources [34] and is likely to result in burnout [35,36]. Moreover, reduced resources owing to FWC may lead to decreased work engagement [37].

Prior studies have linked FWC to negative emotional states including burnout, hostility and guilt [38,39,40,41]. It has also been documented that FWC is negatively associated with work engagement [37]. We therefore hypothesize that:

**Hypothesis** **1a** **(H1a).** *FWC will positively predict burnout.*

**Hypothesis** **1b** **(H1b).** *FWC will negatively predict work engagement.*

### 1.2. Family-To-Work Enrichment

On the contrary, FWE is experienced when the positive facets of employees’ families enhance their work performance [19]. FWE can help individuals to gain resources (e.g., positive experiences, social relationships, or personal skills) in their families and create energy that can be used in their work [20,29,42].

The JD-R model and COR theory may offer explanations for how family-to-work engagement influences safety outcomes. The JD-R model assumes that job resources may elicit a motivational process fostering employees’ development and learning, which in turn can enhance work engagement [30,31]. In addition, employees’ job resources allow them to cope with demands and strain and therefore reduce burnout [30,31]. Considering FWE as a kind of job resources, it is logical to propose that FWE will lead to increased work engagement and decreased burnout.

COR theory [15,16] delineate a human motivation model which argues that acquiring resources is a pivotal human need. Therefore, resources gained from FWE may enhance work engagement. Furthermore, the gain in resources owing to FWE may alleviate the level of burnout.

There has been empirical evidence that FWE can promote work engagement. Siu et al. [42] demonstrated a beneficial impact of FWE on work engagement. Research has also revealed that FWE is negatively associated with burnout [43]. We thus formulate two hypotheses:

**Hypothesis** **2a** **(H2a).** *FWE will negatively predict burnout.*

**Hypothesis** **2b** **(H2b).** *FWE will positively predict work engagement.*

### 1.3. Burnout, Work Engagement, Safety Violations, and Workplace Injuries

Some recent studies have revealed that the interactions between employees’ work and family lives can affect their workplace safety behaviors [10,11]. FWC depletes resources (e.g., time) that people require for maintaining task-related efforts [20,44,45]. Cullen and Hammer [10] found that strain at the work–home interface led to safety violations and low situational compliance. Turner et al. [11] revealed that work–family conflict had an influence on workplace injuries through psychological strain. More generally, Hansez and Chmiel [6] found that under resource loss, people are likely to violate safety rules.

Drawing on the JD-R model and COR theory, we propose that work engagement and burnout may function as mediating variables of the associations of FWC and FWE with workplace injuries. The JD-R model postulates that burnout reduces employees’ mental and physical energy and hence jeopardizes job performance and increases absenteeism, whereas work engagement allows employees to focus their efforts toward goal attainment, resulting in greater work performance and citizenship behavior [8,31]. In terms of workplace safety, burnt-out employees lack the physical and psychological energy to perform safety-focused behavior that prevents them from suffering workplace injuries, whereas engaged employees have higher motivation to follow safety procedures and hence are less likely to injure themselves [6,8,46].

From the perspective of COR theory, as FWC accumulates, employees may experience burnout and decreased work engagement, which in turn may further deplete self-regulatory resources that require employees’ mental effort to regulate their emotions and behaviors to focus on meeting work-related goals [47]. Yet the loss of effective behavior regulation is likely to lead to the violation of safety rules at work. Similarly, Reason et al. [48] suggested that when people put in less effort at work, they are likely to violate their routines and engage in “corner-cutting.” When the violation of routine rules become habitual, actual injuries in the workplace may occur [48]. In contrast, the resources gained from role enrichment may offer instrumental or informational help for goal attainment and thus engage employees in the work domain [8]. As engaged employees feel responsible for their work, they tend to follow safety rules and maintain high safety performance [8].

Studies have indicated that burnout may enhance safety violations and workplace accidents and injuries, while work engagement reduces safety violations and accidents and injuries in the workplace [6,8,46]. Safety violations were operationalized as employees’ tendency to breach safety rules at the workplaces [49] in this study. As safety violations are a predictor of workplace injuries [48,50,51], we expect that safety violations will mediate the impact of burnout and work engagement on workplace accidents. The following hypotheses are formed:

**Hypothesis** **3** **(H3).** *Burnout will positively predict safety violations.*

**Hypothesis** **4** **(H4).** *Work engagement will negatively predict safety violations.*

**Hypothesis** **5** **(H5).** *Safety violations will positively predict workplace injuries.*

**Hypothesis** **6a** **(H6a).** *The relation between FWC and workplace injuries will be mediated by burnout and then safety violations.*

**Hypothesis** **6b** **(H6b).** *The relation between FWC and workplace injuries will be mediated by work engagement and then safety violations.*

**Hypothesis** **6c** **(H6c).** *The relation between FWE and workplace injuries will be mediated by burnout and then safety violations.*

**Hypothesis** **6d** **(H6d).** *The relation between FWE and workplace injuries will be mediated by work engagement and then safety violations.*

Figure 1 illustrates the hypothesized theoretical model.

## 2. Method

### 2.1. Participants and Procedures

We collected research data from nurses and railway workers in Hong Kong using self-administered questionnaires in January 2016 (T1) and seven months later (T2). The seven-month interval was sufficiently long to capture causal effects. The participating nurses were recruited from one local hospital, and the participating railway workers were recruited from one local railway company. All of the measures were administered in Chinese. The research assistants from our research team administered the questionnaires to the target respondents. This study obtained ethical approval from the Office of Research and Knowledge Transfer of Lingnan University.

Among all 525 employees surveyed, 233 (44.4%) completed the questionnaires at 2 time points. Their demographic characteristics are presented in Table 1. A detailed dropout analysis was conducted to compare the demographic characteristics between the responders and non-responders. No significant differences were found in age, χ^2^(7) = 4.79, *p* = 0.686, gender, χ^2^(1) = 1.65, *p* = 0.199, marital status, χ^2^(3) = 4.41, *p* = 0.220, education, χ^2^(3) = 4.28, *p* = 0.233, position, χ^2^(2) = 3.84, *p* = 0.147, shift, χ^2^(1) = 0.51, *p* = 0.475, and tenure, χ^2^(6) = 3.72, *p* = 0.715.

### 2.2. Measures

Apart from the Chinese measures of work engagement and workplace injuries, other originally English measures were translated into Chinese with back-translation to ensure conceptual equivalence. All items of the measures used in this study are presented in the Appendix A.

#### 2.2.1. Family-to-work Conflict (FWC)

To measure FWC, this study adopted the scale constructed by Carlson et al. [52]. The scale has nine items measuring three dimensions: time, behavior and strain. Ratings were completed using a 5-point scale (1 = totally disagree, 5 = totally agree), with greater ratings representing greater FWC.

#### 2.2.2. Family-to-Work Enrichment (FWE)

FWE was assessed with the instrument devised by Carlson et al. [53]. The instrument consists of nine items measuring three subscales: capital, affect, and development. Ratings were given on a 5-point response format (1 = totally disagree, 5 = totally agree), with greater scores representing greater FWE.

#### 2.2.3. Burnout

We employed the Maslach Burnout Inventory (MBI) devised by Maslach et al. [32] to evaluate participants’ levels of burnout. The MBI contains 16 items, with five items measuring emotional exhaustion, five items measuring cynicism, and six items measuring low professional efficacy. Items were scored using a 6-point scale (1 = totally disagree, 6 = totally agree), with greater scores on exhaustion and cynicism and lower scores on professional efficacy reflecting the presence of burnout.

#### 2.2.4. Work Engagement

The Chinese version [20,54] of the Utrecht Work Engagement Scale [55] was utilized to assess how participants engaged with their work. This instrument has nine statements assessing three dimensions of work engagement. There are three statements assessing vigor, three statements assessing dedication, and three statements assessing absorption. All statements were assessed on a 7-point response format (0 = never, 6 = always), with higher numbers indicating higher work engagement. 

#### 2.2.5. Safety Violations

The four-item Bending the Rules Scale [49] was adapted to measures routine safety violations. Ratings were completed on a 5-point scale (1 = totally disagree, 5 = totally agree), with greater scores reflecting greater violations.

#### 2.2.6. Workplace Injuries

To measure workplace injuries, we used the 11-item Chinese measure developed from our previous work [21,22,23]. Each item was evaluated using a 6-point response format (1 = never, 6 = always), with greater ratings reflecting a higher rate of workplace injuries.

### 2.3. Data Analysis

To test the mediation hypotheses, two-wave longitudinal mediation models [56,57] were tested with the structural equation modeling (SEM) approach using Mplus. As our sample was small relative to the number of items, the item parceling technique was applied. For each of the multidimensional measures (FWC, FWE, burnout, and work engagement), three domain-representative parcels were constructed, such that each of the parcels consisted of items from all subscales [58]. For the unidimensional measure of workplace injuries, balanced parcels were constructed based on factor loadings of a single-factor solution [58]. As the measure of safety violations encompassed only four items, these items were employed as the indicators of its latent factor. The measurement model with autocovariances between the error variances of the same observed indicators across the two measurement occasions was evaluated through confirmatory factor analysis (CFA).

**Figure 2:**
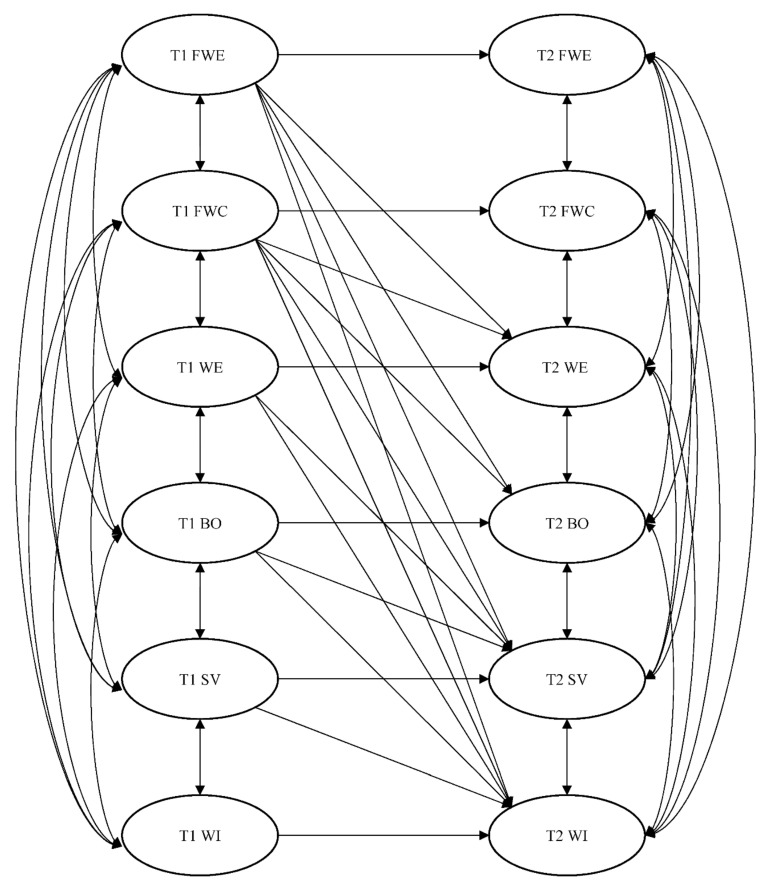
The hypothesized model. FWE = family-to-work enrichment; FWC = family-to-work conflict; WE = work engagement; BO = burnout; SV = safety violations; WI = workplace injuries.

**Figure 3:**
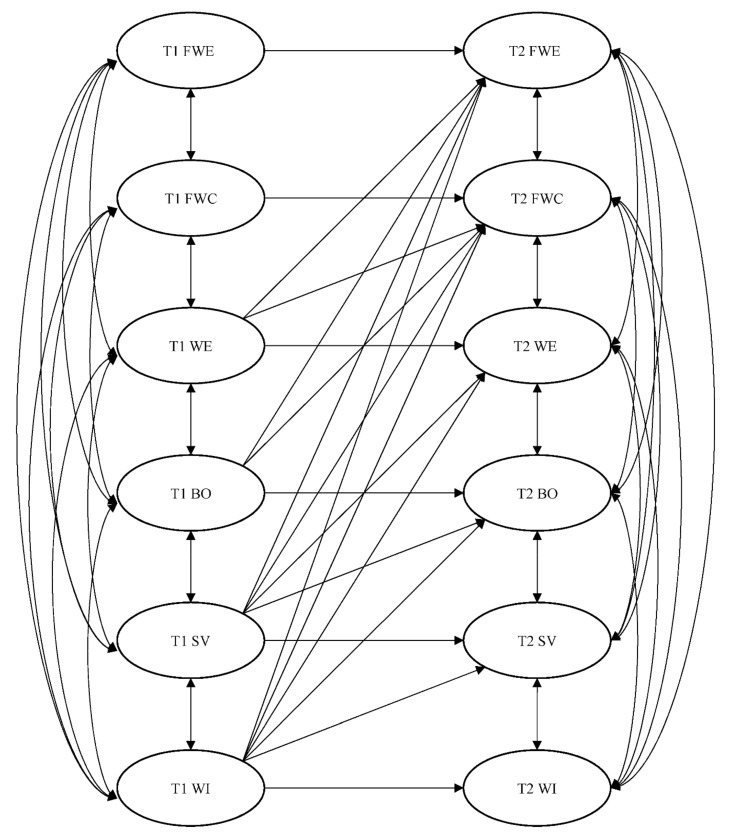
The alternative reversed causation model. FWE = family-to-work enrichment; FWC = family-to-work conflict; WE = work engagement; BO = burnout; SV = safety violations; WI = workplace injuries.

Figure 2 illustrates the hypothesized two-wave longitudinal mediation model. As suggested by [56], two alternative models were also analyzed: (1) a reversed causation model in which the causal directions of the effects were reversed (see Figure 3), and (2) a reciprocal model which incorporated the structural paths specified in the hypothesized model as well as the reversed causation model. All models were evaluated with a combination of fit statistics. In particular, a satisfactory model fit is reflected by RMSEA < 0.06, SRMR < 0.08, CFI > 0.95 and TLI > 0.95 [59,60,61]. For model comparison, chi-square differences (Δχ^2^) were calculated. However, because the reversed causation model and the hypothesized model were not nested, we also used the AIC, which can be applied for comparing nested or a non-nested model [62,63]. A smaller AIC value reflects a better fit. A difference in AIC (ΔAIC) greater than 2.0 indicates a significant difference in model fit [62,64]. The hypothesized mediating effects were tested using the bootstrapping technique. The biased corrected 95% confidence interval (BC 95% CI) was obtained with 2000 bootstrap resamples.

## 3. Results

### 3.1. Descriptive Statistics and the Measurement Model

Descriptive statistics for the major variables are displayed in Table 2. All variables exhibited high internal consistency reliability at T1 (Cronbach’s αs = 0.80–0.93) and T2 (Cronbach’s αs = 0.83–0.94).

CFA showed that the measurement model with autocovariances between the measurement errors across the two occasions fitted the data well, χ^2^(580, *N* = 233) = 824.06, *p* < 0.001, RMSEA = 0.042, 90% CI [0.036, 0.049], SRMR = 0.046, CFI = 0.97, TLI = 0.96. AIC = 13,046.56. All standardized factor loadings were strong, ranging from 0.62 to 0.96.

### 3.2. Hypotheses Testing

The results of SEM indicated that the reciprocal model attained an adequate fit, χ^2^(584, *N* = 233) = 825.51, *p* < 0.001, RMSEA = 0.042, 90% CI (0.035, 0.049), SRMR = 0.047, CFI = 0.97, TLI = 0.96, AIC = 13,040.02. Although the reversed causation model also showed a good fit, χ^2^(597, *N* = 233) = 864.77, *p* < 0.001, RMSEA = 0.044, 90% CI (0.037, 0.050), SRMR = 0.063, CFI = 0.96, TLI = 0.96, AIC = 13,053.28, it was significantly worse than the reciprocal model, Δχ^2^(13, *N* = 233) = 39.26, *p* < 0.001. The hypothesized model exhibited a satisfactory model fit, χ^2^(597, *N* = 233) = 845.62, *p* < 0.001, RMSEA = 0.042, 90% CI (0.036, 0.049), SRMR = 0.054, CFI = 0.97, TLI = 0.96, AIC = 13,034.13, and was not significantly poorer than the reciprocal model, Δχ^2^(13, *N* = 233) = 20.11, *p* = 0.093. Besides, the AIC values revealed that the hypothesized model attained a superior model fit compared with the reciprocal model (ΔAIC = −5.89) and the reversed causation model (ΔAIC = −19.15). The hypothesized model was retained.

**Figure 4:**
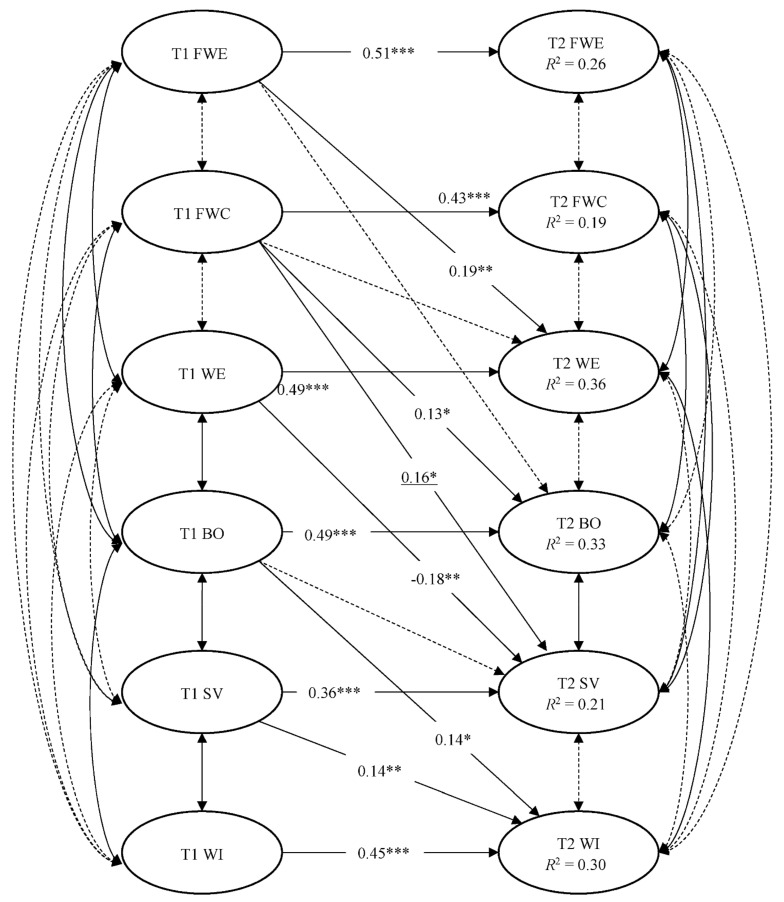
The final model. χ^2^(601, *N* = 233) = 849.42, *p* < 0.001, RMSEA = 0.042, 90% CI (0.035, 0.049), SRMR = 0.055, CFI = 0.97, TLI = 0.96, AIC = 13,029.93. FWE = family-to-work enrichment; FWC = family-to-work conflict; WE = work engagement; BO = burnout; SV = safety violations; WI = workplace injuries. Solid lines represent significant paths and covariances. Dashed lines represent non-significant paths and covariances. Standardized coefficients for significant paths are reported. Observed indicators and autocovariances between measurement errors are omitted for clarity. * *p* < 0.05. ** *p* < 0.01. *** *p* < 0.001.

Following the suggestion by James et al. [65], we further tested a revised model specifying complete mediating effects. Specifically, four non-significant direct effects (from T1 FWE to T2 safety violations and from T1 FWC, T1 FWE, and T1 work engagement on T2 workplace injuries) were dropped from the hypothesized model. The revised model achieved an excellent model fit, χ^2^(601, *N* = 233) = 849.42, *p* < 0.001, RMSEA = 0.042, 90% CI (0.035, 0.049), SRMR = 0.055, CFI = 0.97, TLI = 0.96, AIC = 13,029.93. The removal of these direct effects did not significantly worsen the model fit, Δχ^2^(4, *N* = 233) = 3.80, *p* = 0.434. Besides, the AIC values suggested that the revised model was a better fitting model compared with the hypothesized model (ΔAIC = −4.20). The revised model was accepted as the final model (see Figure 4).

As expected, T1 FWC positively predicted T2 burnout (β = 0.13, *p* = 0.037), whereas T1 FWE positively predicted T2 work engagement (β = 0.19, *p* = 0.003). Hypothesis 1a and 2b were supported. However, T1 FWC did not negatively predict T2 work engagement (β = −0.02, *p* = 0.719), and T1 FWE did not significantly predict T2 burnout (β = −0.10, *p* = 0.060). Hypothesis 1b and 2a were not supported. T1 work engagement negatively predicted T2 safety violations (β = −0.18, *p* = 0.002), whereas T1 burnout did not positively predict T2 safety violations (β = 0.00, *p* = 0.489). These results supported Hypothesis 4 but not Hypothesis 3. Moreover, T1 FWC also positively predicted T2 safety violations (β = 0.16, *p* = 0.037). T1 safety violations positively predicted T2 workplace injuries (β = 0.14, *p* = 0.005), providing support for Hypothesis 5. In addition, T1 burnout also positively predicted T2 workplace injuries (β = 0.14, *p* = 0.013).

The hypothesized indirect effects were examined using the bootstrapping technique. The results are presented in Table 3. According to Cole and Maxwell [56], the product of the three paths from T1 FWE to T2 work engagement, from T1 work engagement to T2 safety violations, from T1 safety violations to T2 workplace injuries represented the indirect effect of FWE on workplace injures via work engagement and safety violations. This indirect effect was significant (*b* = −0.01, BC 95% CI (−0.02, −0.002)), β = −0.01). Hypothesis 6d was supported. Nevertheless, the indirect effect of FWC on workplace injures via burnout and then safety violations (*b* = 0.00, BC 95% CI (−0.004, 0.01)), β = 0.00), the indirect effect of FWC on workplace injures via work engagement and then safety violations (*b* = 0.00, BC 95% CI (−0.003, 0.01)), β = 0.00), and the indirect effect of FWE on workplace injures via burnout and then safety violations (*b* = 0.001, BC 95% CI [−0.01, 0.003]), β = 0.001) were not significant. Hypothesis 6a, 6b, and 6c were not supported.

Furthermore, two additional indirect effects were found. The indirect effect of FWC on workplace injuries via burnout was significant, (*b* = 0.02, BC 95% CI [0.000, 0.07]), β = 0.02). Also, the indirect effect of FWC on workplace injuries via safety violations was significant, (*b* = 0.03, BC 95% CI [0.003, 0.07]), β = 0.02).

## 4. Discussion

This study investigated the mediating roles of burnout, work engagement, and safety violations in the associations of FWC and FWE with workplace injuries. FWC was found to positively predict burnout, while FWE was found to positively predict work engagement. These findings echo past findings that jobs demands engender burnout and job resources engender work engagement [30,31]. Unexpectedly, it was shown that FWC did not negatively predict work engagement, and FWE did not negatively predict burnout. Research has revealed that the impacts of job resources on burnout and the impacts of job demands on work engagement tend to be weak and inconsistent [8,31]. As the JD-R model suggests that burnout is mainly predicted by job demands and work engagement is mainly predicted by job resources [30,31], the effect of FWC on work engagement and the effect of FWE on burnout may become non-significant when the effects of FWC and FWE are taken into account simultaneously.

Work engagement was found to negatively predict safety violations. This finding is in line with past findings [6]. Consistent with prior research [50], we revealed that safety violations positively predicted workplace injuries. Our study further revealed that the association between FWE and workplace injuries was mediated by work engagement and then safety violations. This mediation pathway has not been reported or revealed previously. The finding sheds light onto the mechanism through which FWE influences workplace safety.

Contrary to our prediction, our study found that burnout did not significantly predict safety violations, and the indirect effect of FWC on workplace injuries via burnout and on safety violations was also not significant. However, we found two unanticipated mediating pathways between FWC and workplace injuries. First, burnout significantly mediated the association between FWC and workplace injuries. There has been empirical evidence that burnout could result in workplace injuries [8]. It is plausible that burnout can result in workplace injuries without leading to violations of safety procedures. Second, safety violations significantly mediated the relationship between FWC and workplace injuries. Past investigations have indicated the impact of FWC on safety violations [10]. It is possible that safety violations can result from other consequences of FWC rather than burnout. For example, Cullen and Hammer [10] argued that FWC may create cognitive demands that interfere with employees’ attention to safety protocols. These mediating pathways deserve further investigations in the work–family interface and safety research.

## 5. Implications

This study has valuable contributions. First, we investigated the effects of both the negative (FWC) and the positive (FWE) sides of the family-to-work interface on workplace safety drawing on the JD-R model [30,31] and COR theory [15,16]. It has been established that the work-family interface has both beneficial and harmful influences on employee behavior at work. FWC threatens the quality of both work and familial roles and is perceived as a type of resource loss or potential loss. From the perspective of COR theory [15,16], as resources are valuable, the actual loss or the threat of their loss may result in stress and lead to unsafe behavior. Moreover, work-family enrichment (FWE in this study) helps individuals to gain valued job resources, motivating them to protect their families by maintaining desired performance in their work and family roles, which enhances safety-related behavior. Thus, our research findings extend and go beyond those of Nahrgang et al.’s [8] meta-analysis.

Second, we tested the strain and motivational processes proposed in the JD-R model [30,31], as the underlying psychological mechanisms of safety behaviors. FWC consumes individuals’ valuable resources, especially their self-regulatory resources [66]. The loss of effective regulation of their behaviors is likely to lead to violations of the safety rules at work [67]. FWE helps individuals to gain valuable resources, which is beneficial for employees to meet their work goals. When individuals experience FWE, people under risky or hazardous working conditions may feel more energetic and responsible for complying with safety procedures to protect themselves from being hurt. Hansez and Chmiel [6] provided support for a model that contained the strain and motivational processes as posited by the JD-R model. However, Hansez and Chmiel [6] used other job demands instead of FWC and other job resources instead of FWE. Therefore, our study extends the literature on the JD-R model and workplace safety.

Third, this study revealed the roles of both FWC and FWE in workplace injuries, thus extending the work–family interface framework [13] particularly the family-to-work literature [20]. Our findings offer support that safety violations connect the work–family interface, the strain and motivational processes and safety outcomes. So far, little research on employee safety behavior has simultaneously considered the potential adverse and favorable effects of the family-to-work interface on workplace safety. From the perspectives of the JD-R model [30,31] and COR theory [15,16], the present study investigated the associations of FWC and FWE with employee safety behavior, amalgamating the work-family interface and the psychology of safety arenas.

This study not only contributes to the further development of the research on workplace safety, but also offers practical implications for managing workplace safety by considering work-family interference. In particular, as mentioned earlier, little work has been done on the family-to-work interface. As many workers in high-risk industries perform shift duties (e.g., nurses, railway workers), these professionals are sensitive to work-family interference. As the work-family interface has both favorable and unfavorable effects on employees’ safety performance, organizations must consider ways to alleviate FWC and promote FWE. More intervention workshops on the family domain, such as a supervisor work–family intervention targeting supervisors’ attitudes and skills to help employee manage work–family interface [68], can be conducted in the workplace.

To conclude, the current study has revealed that work engagement and safety violations mediated the relationship between FWE and workplace injuries. Our findings have enriched the theory and research on occupational safety, supporting the call for a fuller and more integrated model incorporating safety, process safety as well and overall employee health as concluded by Hofmann et al. [7] based on their analysis of workplace safety research over 100 years.

## 6. Limitations and Future Directions

Future research is encouraged to further understand the dual process of the work-family interface. This study examined the mediation hypotheses using two waves of data. Future studies are suggested to use a three-wave longitudinal design, which is more accurate in testing indirect effects [56]. Furthermore, the JD-R model points out that job resources and demands could play separate moderating roles. Researchers could test whether this is applicable in the workplace safety domain. Moreover, as our study collected data from nurses and railway workers, future research with employees in diverse occupations is needed to replicate the findings. Finally, we only recruited nurses and railway workers from one local hospital and one railway company. Further studies are suggested to recruit a more representative sample of shift workers.

## 7. Conclusions

This study attempted to advance the existing literature on workplace safety by investigating the mediating effects of burnout, work engagement, and safety violations on the relationships between family-to-work interface (FWC and FWE) and workplace injuries. The results showed that the relationship between FWE and workplace injuries was mediated by work engagement and then safety violations. Burnout mediated the relationship between FWC and workplace injuries. Safety violations also mediated the relationship between FWC and workplace injuries. These findings shed light on the mechanisms underlying the relationship between family-to-work interface and workplace injuries.

## Figures and Tables

**Table 1:** Demographic characteristics of the participants.

	*n*	%
Occupation		
Nurse	166	71.2
Railway worker	67	28.8
Gender		
Male	84	36.4
Female	147	63.6
Age		
24 or below	19	8.3
25–29	58	25.2
30–34	21	9.1
35–39	36	15.7
40–44	26	11.3
45–49	30	13.0
50–54	25	10.9
55 or above	15	6.5
Marital Status		
Never married	123	52.8
Married	110	47.2
Education		
High school or below	185	79.4
Bachelor’s degree or above	48	20.6
Position		
Manager or supervisor	74	31.8
Frontline staff	159	68.2
Shift work		
Yes	142	60.9
No	91	39.1

**Table 2:** Descriptive Statistics for the Major Variables.

Variable	*M*	*SD*	1	2	3	4	5	6	7	8	9	10	11	12
1. T1 FWE	3.58	0.61	(0.93)											
2. T1 FWC	2.52	0.58	0.02	(0.84)										
3. T1 WE	3.19	0.98	0.42 **	−0.04	(0.92)									
4. T1 BO	3.45	0.64	−0.15 *	0.34 ***	−0.25 ***	(0.84)								
5. T1 SV	3.00	0.83	−0.02	0.13	−0.06	0.23 **	(0.83)							
6. T1 WI	1.21	0.38	−0.04	0.03	−0.09	0.18 **	0.17 **	(0.80)						
7. T2 FWE	3.60	0.61	0.50 ***	0.06	0.26 ***	−0.02	−0.07	−0.06	(0.94)					
8.T2 FWC	2.58	0.61	−0.04	0.45 ***	−0.08	0.27 ***	0.20 **	0.01	0.04	(0.88)				
9. T2 WE	3.71	1.12	0.38 **	−0.02	0.58 ***	−0.16 *	−0.07	−0.16 *	0.37 ***	0.00	(0.94)			
10. T2 BO	3.55	0.70	−0.18 **	0.28 ***	−0.23 **	0.56 ***	0.21 **	0.20 **	−0.07	0.39 ***	−0.24 ***	(0.87)		
11.T2 SV	3.19	0.85	−0.09	0.18 **	−0.20 **	0.21 **	0.45 ***	0.05	0.06	0.40 ***	−0.06	0.37 ***	(0.84)	
12. T2 WI	1.27	0.50	0.01	0.13	−0.13 *	0.21 **	0.23 **	0.51 ***	0.00	0.07	−0.22 **	0.18 **	0.13	(0.83)

Note. FWE = family-to-work enrichment; FWC = family-to-work conflict; WE = work engagement; BO = burnout; SV = safety violations; WI = workplace injuries. Values on the diagonals are Cronbach’s α values. * *p* < 0.05. ** *p* < 0.01. *** *p* < 0.001.

**Table 3:** Indirect Effects of Family-to-Work Interface on Workplace Injuries.

Indirect Effect	*b*	BC 95% CI	β
FWC → BO → SV → WI	0.00	[−0.004, 0.01]	0.00
FWC → WE → SV → WI	0.00	[−0.003, 0.01]	0.00
FWE → BO → SV → WI	0.00	[−0.01, 0.003]	0.00
FWE → WE → SV → WI	−0.01 *	[−0.02, −0.002]	−0.01
FWC → BO → WI	0.02 *	[0.000, 0.07]	0.02
FWC → SV → WI	0.03 *	[0.003, 0.07]	0.02

Note. FWC = family-to-work conflict; FWE = family-to-work enrichment; BO = burnout; WE = work engagement; SV = safety violations; WI = workplace injuries; BC 95% CI = Biased corrected 95% confidence interval. BC 95% CIs are obtained with 2000 bootstrap resamples. * *p* < 0.05.

## Data Availability

No additional data are available.

## Appendix A

Items of the Measures Used in this Study

Family-to-work Conflict [52]

1. The time I spend on family responsibilities often interferes with my work responsibilities.
2. The time I spend with my family often causes me not to spend time in activities at work that could be helpful to my career.
3. I have to miss work activities due to the amount of time I must spend on family responsibilities.
4. Due to stress at home, I am often preoccupied with family matters at work.
5. Because I am often stressed from family responsibilities, I have a hard time concentrating on my work.
6. Tension and anxiety from my family life often weakens my ability to do my job.
7. The behaviors that work for me at home do not seem to be effective at work.
8. Behavior that is effective and necessary for me at home would be counterproductive at work.
9. The problem-solving behavior that works for me at home does not seem to be as useful at work.

Family-to-work enrichment [53]

My involvement in my family:

1. Helps me gain knowledge and this helps me to be a better worker
2. Helps me acquire skills and this helps me to be a better worker
3. Helps me expand my knowledge of new things and this helps me to be a better worker.
4. Puts me in a good mood and this helps me to be a better worker
5. Makes me feel happy and this helps me be to be a better worker
6. Makes me cheerful and this helps me to be a better worker
7. Requires me to avoid wasting time at work and this helps me to be a better worker
8. Encourages me to use my work time in a focused manner and this helps me to be a better worker.
9. Causes me to be more focused at work and this helps me to be a better worker

Work Engagement [55]

1. At my work, I feel bursting with energy.
2. At my job, I feel strong and vigorous.
3. I am enthusiastic about my job.
4. My job inspires me.
5. When I get up in the morning, I feel like going to work.
6. I feel happy when I am working intensely
7. I am proud of the work that I do.
8. I am immersed in my work.
9. I get carried away when I am working.

Burnout [32]

1. I feel mentally exhausted by my work.
2. I doubt the purpose of my work.
3. An entire day of work is a great toll for me.
4. I can solve the problems in my work adequately.
5. I feel “burned out” by my work.
6. I have the feeling that I contribute positively with my work at the functioning of the organization.
7. I see that I distanced myself from my work too much.
8. I’m not as enthusiastic as I used to be about my work.
9. I think I’m doing my work well.
10. If I finish something at work, I become happy.
11. At the end of a workday I feel empty.
12. I achieved a lot of valuable things in this job.
13. I just want my job not being bothered any further.
14. I feel tired when I get up in the morning and there is another workday ahead of me.
15. I became more cynical about the effects of my work.
16. At work, I have a lot of self-confidence.

Safety Violations [49]

1. I sometimes cut corners if it makes the task easier.
2. Production pressures mean that I sometimes bend the rules.
3. Occasionally I bend the rules when I know it is safe to do so.
4. When my boss is not around, I can be more flexible with which procedures I follow.

Workplace Injuries [23]

Over the past 6 months, how often have you had the following injuries during work?

1. sprains/strains
2. scratches/cuts/punctures
3. burns
4. contusion/struck by objects
5. bone crack/broken
6. bone dislocation
7. Slip/trip/fall
8. injured when moving items/objects
9. injured when doing some movements
10. injured when using tools
11. injured in collisions at work
